# Differences in Disease Severity but Similar Telomere Lengths in Genetic Subgroups of Patients with Telomerase and Shelterin Mutations

**DOI:** 10.1371/journal.pone.0024383

**Published:** 2011-09-13

**Authors:** Tom J. Vulliamy, Michael J. Kirwan, Richard Beswick, Upal Hossain, Charlotte Baqai, Anna Ratcliffe, Judith Marsh, Amanda Walne, Inderjeet Dokal

**Affiliations:** 1 Centre for Paediatrics, Barts and The London School of Medicine and Dentistry, Queen Mary University of London, London, United Kingdom; 2 Department of Haematology, Imperial College, Hammersmith Hospital, London, United Kingdom; 3 Department of Haematological Medicine, King's College Hospital, London, United Kingdom; University of Minnesota, United States of America

## Abstract

The bone marrow failure syndrome dyskeratosis congenita (DC) has been considered to be a disorder of telomere maintenance in which disease features arise due to accelerated shortening of telomeres. By screening core components of the telomerase and shelterin complexes in patients with DC and related bone marrow failure syndromes we have identified 24 novel mutations: 11 in the RNA component of telomerase (*TERC),* 8 in the reverse transcriptase component (*TERT)*, 4 in dyskerin (*DKC1)* and 1 in TRF1-interacting nuclear factor 2 (*TINF2*). This has prompted us to review these genetic subtypes in terms of telomere length, telomerase activity and clinical presentation among 194 genetically characterised index cases recruited onto the registry in London. While those with *DKC1* and *TINF2* mutations present at a younger age and have more disease features than those with *TERC* or *TERT* mutations, there is no difference in telomere length between these groups. There is no difference in the age of onset and numbers of disease features seen in those with *TERC* and *TERT* mutations despite the fact that the latter show higher levels of telomerase activity *in vitro*. The incidence of aplastic anaemia is greater in patients with *TERC* or *TINF2* mutations compared to patients with *DKC1* mutations, and cancer incidence is highest in patients with *TERC* mutations. These data are the first to provide robust comparisons between different genetic subtypes of telomerase and shelterin mutations (the “telomereopathies”) and clearly demonstrate that disease severity is not explained by telomere length alone.

## Introduction

Telomeres are nucleoprotein structures that protect chromosome ends, distinguishing them from double strand breaks that might occur elsewhere in the genome and need repair [1]. Telomeric DNA consists of a TTAGGG repeat sequence that is bound by a protein complex known as shelterin [2]. Because of the end-replication problem [3] telomeres shorten with each cell division and when they become critically short, a p53-dependent checkpoint is activated that leads to apoptosis or cell senescence [4]–[6]. It has been suggested that this mitotic clock has a tumour-suppressor function in long lived mammals, giving cells a limited lifespan and hence restricting the accumulation of DNA damage [7]. Telomere shortening has therefore been linked to the processes of both aging [8] and cancer [9]. In the germ line and in some stem cells, telomeric DNA can be replenished by telomerase, a ribonucloeprotein complex that involves an RNA template and a reverse transcriptase [10].

Dyskeratosis congenita (DC) is an inherited multi-system disorder classically characterized by a mucocutaneous triad of abnormal skin pigmentation, nail dystrophy and leukoplakia [11]. DC patients frequently develop bone marrow failure and are at a high risk of developing cancer [12] as well as a variety of other features [13], [14]. DC is clinically and genetically heterogeneous. Eight disease genes (*DKC1, TERC, TERT, NOP10, NHP2, TINF2, C16orf57* and *TCAB1)* have been identified to date [15]–[22] and all but one are known to be involved in telomere maintenance. The four most common genetic subtypes of DC are those that involve either the core components of the telomerase complex [23], the RNA (*TERC)*, the reverse transcriptase (*TERT*) and the accessory protein dyskerin (*DKC1),* or the shelterin component, the telomeric repeat binding factor 1-interacting nuclear factor 2 (*TINF2).*

It is not surprising therefore that DC has been considered to represent the clinical manifestation of defective telomere maintenance and indeed, very short telomeres are a hallmark of this disease [24]–[26]. However, there is a broad clinical spectrum associated with these mutations and patients can present with a number of overlapping disorders, characterised by one or more of a constellation of clinical features [7], [27]. In addition to DC, these include the Hoyeraal-Hreidarsson (HH) syndrome [28], which is characterised by a variety of features including bone marrow failure, growth retardation, cerebellar hypoplasia, enteropathy and immunodeficiency. Other patients may present with only aplastic anaemia (AA) [29], [30], myelodysplastic syndrome (MDS) [31], [32], acute myeloid leukaemia (AML) [33] pulmonary fibrosis [34], [35] and liver fibrosis [36], and collectively these can be referred to as the ‘telomereopathies’.

We have been screening telomere-related genes among patients referred, primarily with bone marrow failure, to our registry in London and in this paper we report on 24 novel mutations. The variable clinical presentation among these patients has prompted us to review the clinical features, telomere length and mutation status among 194 genetically characterised index cases. While there are clear differences between the genetic subgroups in terms of clinical presentation, we find that the range of telomere length is similar in each group. We conclude that telomere length alone does not account for variation in disease severity between the different genetic subtypes.

## Results

### Novel mutations in telomerase and shelterin components

Through mutation screening in patients with DC and related bone marrow failure syndromes, we have identified 23 novel mutations in core components of telomerase: 11 in *TERC*, 8 in *TERT* and 4 in *DKC1* (Table 1, Figures S1 and S2). Two additional *TERT* mutations were identified during the course of this study which have been reported elsewhere in unrelated families [15], [37]. One mutation in the shelterin component *TINF2* has been identified (reported also by Sasa et al [38] while this paper was in preparation) which is in addition to the 8 *TINF2* mutations that we have recently reported elsewhere [39]. All *TERC, TERT* and *TINF2* mutations were heterozygous, while the *DKC1* mutations are hemizygous (X-linked). None have been reported previously and none are reported among previous screens of normal healthy individuals [15], [16], [20], [30] or on dbSNP or the 1000 genomes database. The clear majority (8/11) of the *TERC* mutations disrupt base pairing in the pseudoknot region of the molecule and they look like classic disease causing mutations (Figure S1). Of all 45 disease-associated *TERC* mutations that are known to us, 30 are located in the pseudoknot. Also consistent with previous findings [30], [36], [37], the *TERT* mutations are spread throughout the molecule and show variable degrees of conservation across species (Figure S2). Polyphen conservation scores support the notion that most of these mutations are disruptive: 8/10 are predicted to be probably damaging (score = 0.949−1.0) and one is possibly damaging (Val56Leu, score = 0.433); the only one predicted to be benign (Arg972His, score = 0.059) is seen in combination with a second substitution, Gly1063Asp, which has been reported in another patient elsewhere [37].

**Table 1:** Characteristics of patients with novel telomerase and shelterin mutations.

Age (years)	Disease^1^	Additional disease features^2^	FH^3^	Relatives^4^	Nucleotide	Location^5^ or amino acid	TRAP^6^	Δtel^7^
***TERC***								
18	CAA	dysphagia, skin pigmentation, low NK cells	No	AsM	36C>T	Ps P1b	11.3	
22	CAA	skin pigmentation	No		67G>A	Ps P2a.1	4.6	
24	AA/MDS	non-cirrhotic portal hypertension with oesophageal varices	No		83T>G	Ps P2a	4.3	−2.52
40	DC	dental & hair loss, pulmonary disease, cancer	No	AsM, AsSo	95_96delGC	Ps P3	0.6	−2.82
29	CAA	microcephaly, aseptic necrosis of femoral head	No	AsF	107G>T	Ps P3	0.9	−4.30
28	NSAA		No		126A>G	Ps P2a	4.8	−6.22
34	AA	aseptic necrosis of femoral head	No	AsSo	176A>C	Ps P3	0.5	−3.58
18	CAA	nail dystrophy	No		182G>A	Ps P3	1.3	−3.03
5	HH	microcephaly, cerebellar hypoplasia, hair loss, low NK cells	No	AsM, AsSi	242C>T	Loop P5	46.8	−0.57
55	MDS	mucocutaneous features, lung disease, abnormal LFT	No		287C>G	CR4-CR5 P6b	8.1	−0.57
31	CAA	skin pigmentation, VSD	Yes	S?	377A>G	H box		
***TERT***								
14	NSAA	easy fracture	No	AsM	166G>C	Val56Leu	4.3	−3.48
26	AA/MDS	premature greying, telangiectasia	Yes	SF, SGm	248G>C	Arg83Pro		
5	DC		No		1142G>C	Arg381Pro	43.1	
12	CAA	short digit	Yes	AsF, Ssi	2147C>T	Ala716Val^8^	16.6	−3.14
48	DC	MDS, IPF, hair loss, dental caries	Yes	SB, AsSi	2152G>A	Asp718Asn	44.1	−3.61
25	MDS	liver cirrhosis, congenital hearing loss, bilateral nystagmus	No	AsM	2581G>A	Gly861Arg	1.0	
11	DC	hyperhiderosis	Yes	SB, SB	2915G>A	Arg972His		
19	DC	hypogonadism	No		3082A>C	Asn1028His	16.7	−2.20
11	DC	hyperhiderosis	Yes	SB, SB	3187G>A	Gly1063Ser^8^	29.0	
34	NSAA	abnormal LFT	No		3388A>G	Ile1130Val		
***DKC1***								
1	HH	microcephaly, cerebellar hypoplasia, low B cells	Yes	SB	202C>T	His68Tyr		
15	DC		No		227C>T	Ser76Leu		−2.66
4	HH	microcephaly, enteropathy, immunodeficiency	No		1133G>A	Arg378Gln		−0.7
19	DC		No		114C>G	Ile38Met		
***TINF2***								
6	AA	Intracranial calcification, ocular hemorrhage	No		811C>T	Gln271X		

1 AA: aplastic anaemia, CAA: constitutional AA, NSAA: non-severe AA, MDS: myelodysplastic syndrome, HH: Hoyeraal Hreidarrson syndrome.

2 NK: natural killer cells; VSD: ventricular septal defect; IPF: idiopathic pulmonary fibrosis; LFT: liver function test.

3 FH: family history.

4 Relatives are listed where they were available and tested positive for the mutation. As = asymptomatic, S = symptomatic, M = mother, F = father, So = son, Si = sister, B = brother, Gm = grandmother.

5 Location: domain of TERC: Ps = pseudoknot.

6 TRAP: telomere repeat amplification protocol, % wild type.

7 Δtel: age-adjusted telomere length measurement.

8 also published in another family elsewhere [15], [37].

There is a broad spectrum of clinical presentation among these patients, ranging from early onset of features resembling the HH syndrome to a patient presenting at the age of 55 years with MDS. It is noteworthy that of the 21 index cases with *TERC* or *TERT* mutations only 7 had sufficient clinical features to be classified as DC or HH; 14 out of the 21 were classified as having either idiopathic AA, constitutional AA or MDS. Another striking observation here is that of the patients with *TERC* mutations, only one reports that a family member had presented with any features related to the disease (Table 1, column ‘FH’). A lack of family history is normal among patients with *TINF2* mutations due to the fact that these mutations usually arise *de novo*. However, this is not the case for the *TERC* families: when no family history is reported and both parents are available, one of these parents is usually an asymptomatic carrier.

### *TERC* mutations cause lower telomerase activity than *TERT* mutations

**Figure 1:**
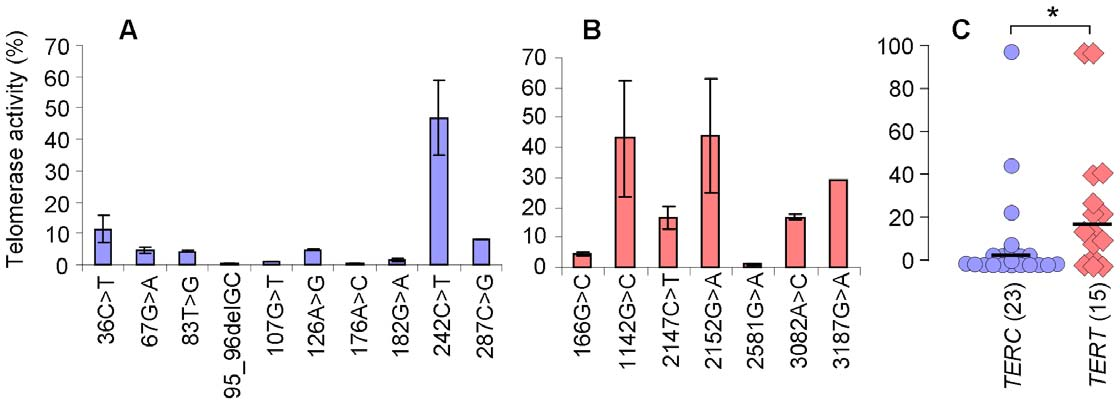
*TERC* mutations have lower residual telomerase activity than *TERT* mutations. Telomerase activity of (**A**) novel *TERC* mutations and (**B**) novel *TERT* mutations were determined using the TRAP assay and are expressed as a % of wild-type activity. (**C**) A comparison of all *TERC* mutations (n = 23, blue circles) and *TERT* mutations (n = 15, pink diamonds) that we have studied shows that as a group, the *TERC* mutations give significantly lower residual telomerase activity (* P value = 0.04). Black bars indicate median values.

The *in vitro* telomerase activity of the *TERC* and *TERT* mutations, as determined by the telomere repeat amplification protocol (TRAP assay) may act as a guide to the clinical penetrance of these different alleles. It is noticeable that for all but one of the *TERC* mutations, the TRAP activity is <12% of wild type (Table 1, Figure 1A). The exception is the *TERC* 242C>T mutation, which gives an activity of 47±11%; this mutation is located in a loop bordering the CR4-CR5 domain of the molecule, away from other disease-causing alleles. It is seen in a patient who has HH, consanguineous parents, and whose asymptomatic mother and sister are also heterozygous; this may therefore not be the pathogenic lesion in this family.

In contrast, only 2/7 of the *TERT* mutations give a TRAP activity of <10% wild type, while the others give an activity >15% normal (Table 1, Figure 1B). When we include mutations that we have analysed previously, (14 in *TERC* and 8 in *TERT*), we see that as a group, the *TERC* mutations give significantly lower telomerase activity than the *TERT* mutations (Figure 1C, Student’s t-test P-value = 0.04).

### Disease severity varies between different genetic subtypes

**Figure 2:**
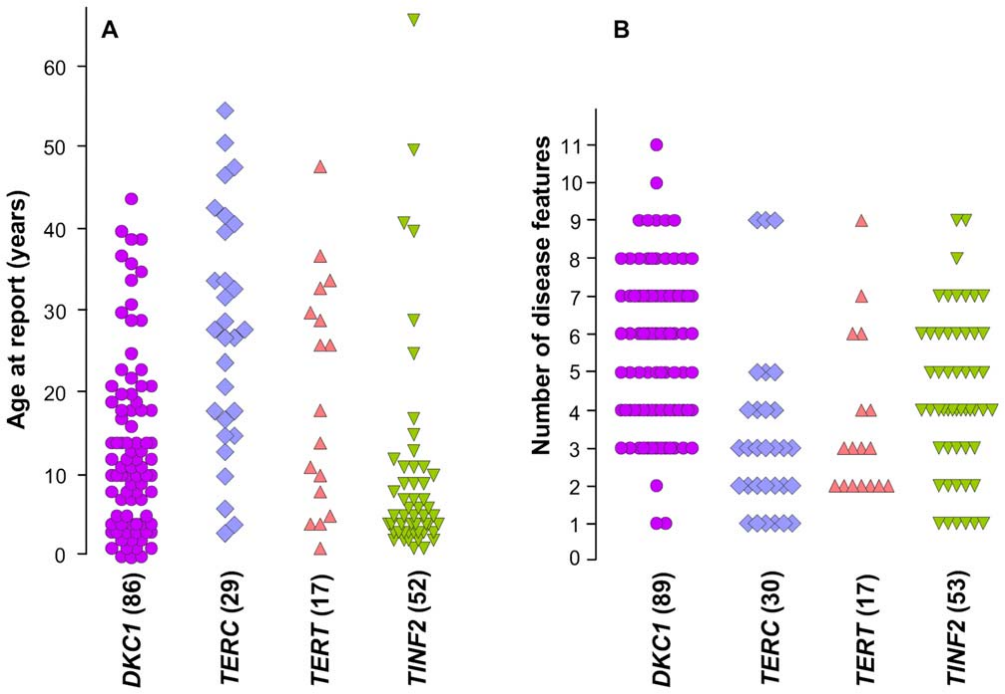
Differences in clinical presentation between genetic subgroups of patients. (**A**) Age at report of patients with different telomerase or shelterin mutations, as indicated below the panel. (**B**) The number of disease features observed in the same group of patients. The numbers of subjects (in brackets) differ slightly, reflecting the fact that some information is missing. The disease features that were scored included: abnormal skin pigmentation, nail dystrophy, leucoplakia, AA/cytopenia, immunodeficiency, cancer, oesophageal stenosis/dysphagia, enteropathy, liver disease, splenomegaly, pulmonary disease, epiphora, ear abnormality, deafness, dental decay/loss, hair greying/loss, retinopathy, microcephaly, cerebellar hypoplasia/ataxia, learning disabilities/developmental delay, abnormal facies, intrauterine growth retardation/low birth weight, short stature/growth retardation, aseptic necrosis, osteoporosis, renal disease, gonadal abnormality, phimosis, hyperhiderosis and cardiac disease.

These novel data have prompted us to review the clinical and biological manifestations of different mutations across a large series of patients referred to our registry in London. We have information relating to 194 index cases in which we have defined the genetic basis of the disease: 91 with *DKC1,* 56 with *TINF2,* 30 with *TERC* and 17 with *TERT* mutations. As indicators of disease severity, we have compared the age of report and the number of presenting disease features between the different subtypes (Figure 2). No difference is seen in either category between patients with *TERC* and *TERT* mutations. However, those with *DKC1* mutations present at a significantly younger age compared to those with *TERC* mutations (Student's t-test, P-value <.0001) and have a significantly greater number of disease features than those with both *TERC* and *TERT* mutations (P-value <.0001). Those with *TINF2* mutations present at a younger age than those with both *TERC* and *TERT* mutations (P-value <.0001 and 0.008, respectively) and have more disease features than those with *TERC* mutations (P-value = 0.027).

We have also investigated the relative incidence of several clinically significant disease features: the presence of 2 or more of the classical mucocutaneous features, AA, cancer, pulmonary disease, cerebellar hypoplasia and short stature (Table 2). Again, no difference is seen in any category between patients with *TERC* and *TERT* mutations. However, mucocutaneous features are far less common in patients with either *TERC* or *TERT* mutations compared to *DKC1* (Pearson's chi-squared test, P-value <0.0001). Patients with *TINF2* mutations are also less likely to have mucocutaneous features than the *DKC1* patients (P-value = 0.006), but are more likely to have them than in either *TERC* or *TERT* patients (P-value = 0.014 and 0.019, respectively). The incidence of AA is less among patients with *DKC1* mutations than either *TERC* or *TINF2* patients (P-value  = 0.007 and 0.006, respectively); this is despite the fact that the *TINF2* patient group is younger than the *DKC1* group, while the *TERC* group is older than the *DKC1* group. Cancer incidence is higher among patients with *TERC* and *TERT* mutations, significantly so compared to the *TINF2* group (P-value  = 0.003 and 0.011, respectively); this may well reflect the fact that they are generally an older patient group.

**Table 2:** Number of index cases with different disease features amongst different genetic subtypes.

Gene	Number of cases	Aplastic anaemia (%)	2 or more mucocutaneous features (%)	Cancer (%)	Pulmonary disease (%)	Cerebellar hypoplasia (%)	Short stature (%)
*DKC1*	91	44 (48.4)	60 (65.6)	7 (7.7)	6 (6.6)	15 (16.5)	19 (20.8)
*TINF2*	56	40 (71.4)	24 (42.9)	1 (1.8)	3 (5.5)	6 (10.7)	12 (21.4)
*TERC*	30	23 (76.7)	5 (16.7)	6 (20.0)	4 (13.3)	1 (3.3)	3 (10)
*TERT* *	17	12 (70.6)	2 (11.8)	3 (17.6)	3 (17.6)	1 (5.8)	2 (11.8)
All	194	119 (61.3)	91 (46.9)	17 (8.8)	16 (8.3)	23 (11.9)	36 (18.6)

*Homozygotes excluded.

### Telomere lengths do not explain differences in disease severity between genetic subtypes

**Figure 3:**
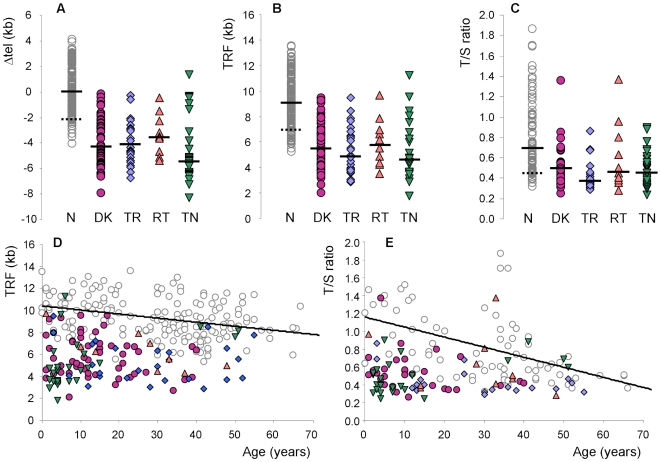
Telomere length measurements in patients with telomerase and shelterin mutations. Different genetic subgroups of patients with telomerase and shelterin mutations, shown as purple circles (*DKC1* mutations), blue diamonds (*TERC* mutations), pink triangles (*TERT* mutations) and inverted green triangles (*TINF2* mutations) are compared to healthy controls (open grey circles). Solid bars indicate median values and the dotted bars indicate the 10^th^ centiles of the healthy control measurements. (**A**) Telomere lengths were measured by Southern blot analysis and adjusted for age to give a Δtel value (54). N = normal healthy controls (n = 176), DK = patients with *DKC1* mutations (n = 56), TR = patients with *TERC* mutations (n = 26), RT = patients with *TERT* mutations (n = 10), TN = patients with *TINF2* mutations (n = 26). (**B**) Absolute telomere lengths, measured as terminal restriction fragments for the same group of patients as shown in panel A. (**C**) Telomere lengths measured as a T/S ratio, determined by monochrome multiplex quantitative PCR for a subset of patients shown in panel A (73 healthy controls and 29, 19, 10 and 23 patients with *DKC1, TERC, TERT* and *TINF2* mutations, respectively). (**D**) Absolute telomere lengths (terminal restriction fragments) plotted against age in years, for the patients and health controls shown in panel A. A line of best fit is drawn for the healthy controls. (**E**) Telomere lengths measured by monochrome multiplex quantitative PCR analysis plotted against age in years for all patients in panel C. A line of best fit is drawn for the healthy controls.

Despite the fact that we see significant differences in clinical severity between the different genetic subtypes of patients, both in terms of age of presentation and the number of disease features, we do not see any difference in telomere lengths between these groups of index cases. This is true whether we compare age adjusted (Figure 3A) or absolute (Figure 3B) telomere lengths as measured by Southern blot analysis, or when we compare telomere lengths determined by quantitative PCR (Figure 3C). Plots of telomere length against age (Figure 3D and 3E) are consistent with the idea that disease presents when telomeres shorten below a certain length, independent of age.

**Figure 4:**
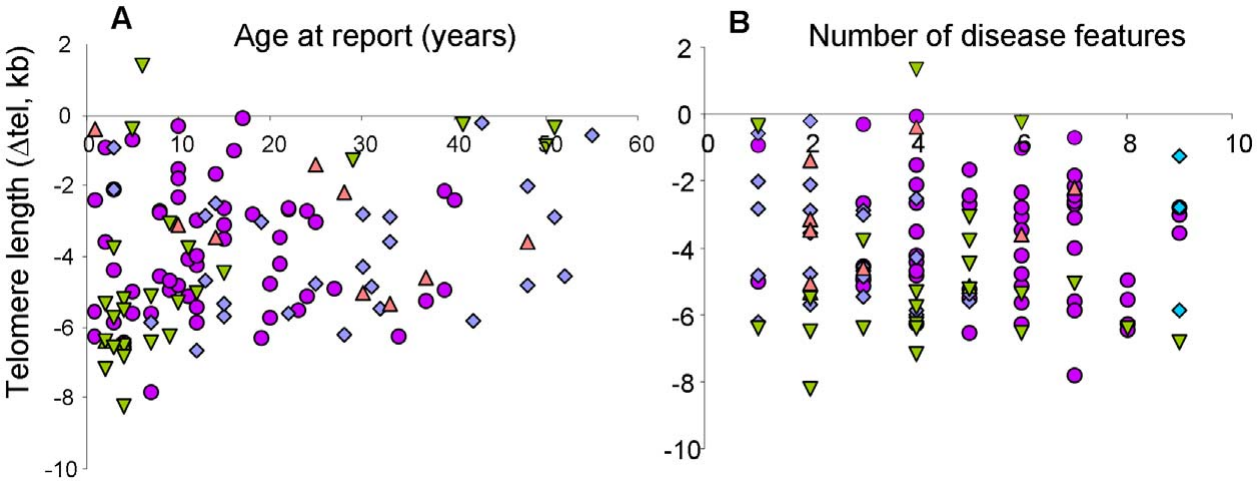
No correlation between telomere length and clinical presentation. Among all index cases (n = 118) there is no correlation seen between telomere length and either (**A**) the age at report (R^2^ = 0.062) or (**B**) the number of disease features observed (R^2^ = 0.007). Symbols for the different disease subgroups are as described for Figure 3.

**Figure 5:**
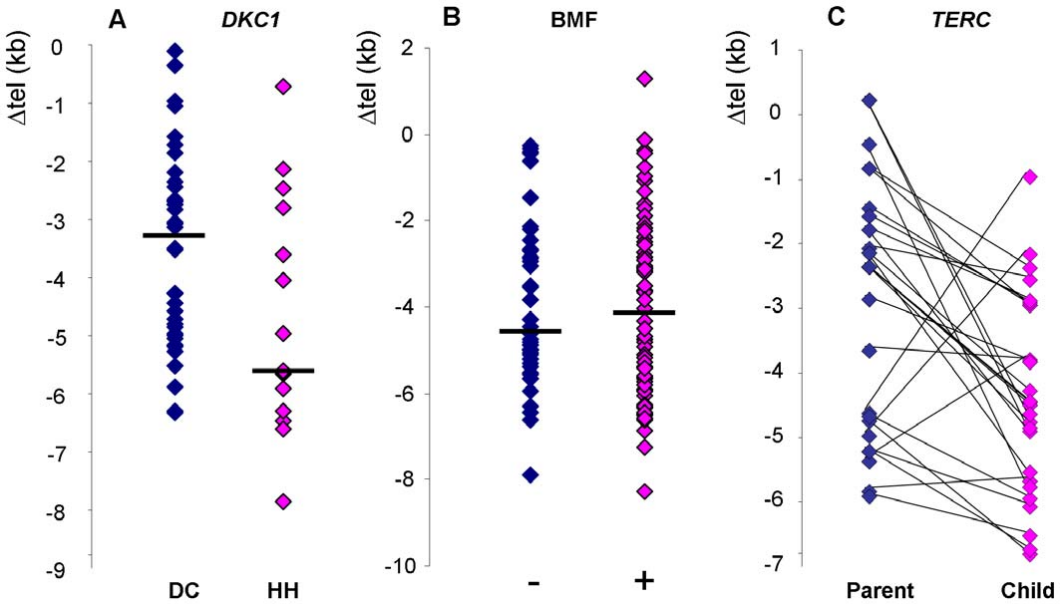
Comparison of telomere lengths in specific patient subgroups. (**A**) Amongst patients with *DKC1* mutations, telomere lengths are longer in those that have the more classical disease, DC (n = 42, indigo diamonds), compared to those that have the more severe HH phenotype (n = 14, pink diamonds). (**B**) No difference in telomere length is seen between patients who presented with bone marrow failure (n = 66, pink diamonds) and those that did not have bone marrow failure (n = 46, indigo diamonds). (**C**) In 27 parent-child combinations of subjects with heterozygous *TERC* mutations, the children (with 4 exceptions) have shorter telomeres than their parents.

Taking all index cases together, there is no correlation between telomere length and either age of report (Figure 4A) or number of disease features (Figure 4B). The same is true if each subtype is analysed separately (Figures S3 and S4), although there is a trend toward earlier presentation with shorter telomeres among patients with *TINF2* mutations (R^2^ = 0.424). However, we do see that among patients with *DKC1* mutations, those that present with the severe HH syndrome have shorter telomeres than those that present with more classical DC (P-value = 0.03, Figure 5A). Somewhat surprisingly, when we compare all patients who did and did not present with bone marrow failure, we do not see any difference in telomere length (Figure 5B).

Another factor that can be taken into consideration is the number of asymptomatic relatives (both younger and older) that exist in each of the subtypes: while we know of very few asymptomatic relatives with *TINF2* or *DKC1* mutations, this is a common feature of families with *TERC* and *TERT* mutations. It has been proposed that is at least in part due to disease anticipation in families with both *TERC* and *TERT* mutations, and that this is associated with progressive telomere shortening through the generations. We now know of 27 parent-child combinations from 15 different families in which both are *TERC* heterozygotes. In all but 4 of these combinations, the affected child has a shorter age-adjusted telomere length than their affected parent (Figure 5C). As a group, the children have significantly shorter telomeres than the parents (P value = 0.0026) with a median difference in Δtel between parent and child (Δtel, child-Δtel, parent) of −1.53 kb.

## Discussion

This is the first study to systematically review the relationship between telomere length and disease severity in a large cohort of unrelated patients with mutations in different telomere-related genes. While clear phenotypic differences exist between the genetic subtypes, there is no difference in the range of telomere lengths observed. This conclusion may seem somewhat surprising as several previous studies have indicated that disease severity may be related to telomere length. These include the phenomenon of disease anticipation associated with telomere shortening in families with *TERC* and *TERT* mutations [17], [40], [41], shorter telomeres among patients with *TINF2* mutations [42], and patients with severe disease caused by *DKC1* mutations having shorter telomeres than those with mild disease [43].

There are several differences between previous studies and the one we present here. Firstly, we are now looking at a considerably larger number of patients with telomere defects, representing the largest series published to date. Secondly, we have focused on the index cases only to avoid the issues relating to asymptomatic individuals with telomerase/shelterin mutations, particularly in the context of disease anticipation. Thirdly, we are comparing different genetic subtypes of the disease, rather than looking within a single subtype.

The four common genetic subtypes of DC (caused by mutations in the *DKC1, TINF2, TERC,* and *TERT* genes) can be divided into three groups: (i) the inherited autosomal dominant mutations in the telomerase-specific components, *TERC* and *TERT;* (ii) the X-linked recessive mutations in *DKC1,* encoding a protein that acts as a pseudouridine synthase in H/ACA small nuclear ribonucleoprotein (snoRNP) complexes [44], predominantly involved in ribosomal RNA processing [45], as well as a core component of telomerase; and (iii) the predominantly *de novo* mutations in *TINF2,* encoding a core component of the telomere-binding protein complex, shelterin [46], [47]. Mutations in any of these genes can clearly impact dramatically on the ability to maintain telomere length and result in a multisystem disease. However, the fact that three different pathways are affected in the different genetic subtypes may explain why telomere length alone does not correlate with disease severity. This suggests other biological defects are likely to be important in the disease pathology and the resulting overall clinical phenotype. Although there is no clear evidence of a defect in pseudouridylation in patients with *DKC1* mutations [48], [49], several studies have indicated that in mice they can have a significant impact beyond telomere maintenance, affecting snoRNA accumulation [50], the DNA damage response [51] and IRES-mediated translation [52].

No significant differences were observed in clinical presentation of patients with *TERC* and *TERT* mutations in our cohort. This is despite the fact that as a group, the *TERC* mutations gave lower residual telomerase activity than *TERT* mutations in the *in vitro* TRAP assay. No equivalent comparison has been made previously although a trend toward higher residual TRAP activity of *TERT* mutations has been apparent in other studies [26], [30], [32]. In the largest series of *TERT* mutations published to date [37], the mean age of 134 carriers was 51 years; 50% of these heterozygotes had pulmonary disease but only 16% had haematological abnormalities. This highlights the variable penetrance as well as the diverse and heterogeneous nature of the disease caused by *TERT* mutations. A similar spectrum of disease severity can be observed in families with *TERC* mutations, although these appear more likely to present with haematological abnormalities than pulmonary disease. Nevertheless, the disease seen in patients with both *TERC* and *TERT* mutations clearly contrasts dramatically with the severe early onset of DC, the HH syndrome, and/or AA that can be seen in patients with mutations in either the shelterin component, *TINF2* or the snoRNP component, *DKC1.*

It is interesting to consider how the rate of telomere shortening differs between the genetic subtypes, even though this is very difficult to measure empirically in individual patients. However, in a typical patient with a *TINF2* mutation (e.g. the recurrent Arg282His substitution), this will have arisen *de novo* and presentation, with very short telomeres occurs in the early years of life. A similar rapid shortening of telomeres will have occurred in approximately 1/3 of patients with *DKC1* mutations (e.g. the recurrent Ala353Val substitution) in which the mutation will also have occurred *de novo*; in patients that inherit a *DKC1* mutation, they do so from an asymptomatic heterozygous mother, who will show a highly skewed pattern of X-inactivation in peripheral blood. Both situations are completely different to the typical presentation of *TERC* and *TERT* mutations which, although dominant, are usually present in the family for at least one asymptomatic generation. The rate of telomere shortening here appears to be much slower, with critically short telomeres manifesting in disease at an older age and after transmission through the germ line.

One confounding factor in the comparison of different mutations within and between genetic subtypes is the diversity of the mutations themselves. Some mutations are clearly and recognizably pathogenic because they recur (such as Ala353Val in *DKC1* and Arg282His in *TINF2)* or because they abolish telomerase activity (as seen in *TERC* and *TERT).* However with private missense mutations in *DKC1* and *TINF2* or with mutations that only partially reduce telomerase activity in *TERC* and *TERT,* it is more difficult to be sure that they are disease causing, rather than bystander mutations.

The significant clinical differences observed between the genetic subgroups (such as the early onset of disease and greater number of disease features in *DKC1* and *TINF2* patients compared to *TERC* and *TERT* patients and the higher incidence of cancer in patients with *TERC* mutations) will facilitate more informed decisions in the clinic. Equally these findings serve to highlight that whilst these genetic subtypes are unified by having a defect in telomere biology (the “telomereopathies”) the clear differences they exhibit suggest other factors (inherited and acquired) contribute to the overall clinical phenotype. Finally, as a major subgroup of the novel mutations were observed in patients who did not fulfil the criteria to be classified as DC, this study highlights the importance of screening the telomerase and shelterin genes in patients with a spectrum of clinical phenotypes in addition to DC.

## Methods

### Subjects

All individuals included in this study have given written informed consent in accordance with the Declaration of Helsinki and our study design, approved by the East London and The City Research Ethics Committee. The novel mutations reported in this study were identified among 732 patients referred to our registry over a period of 69 months. They comprised 318 patients with AA, 99 patients who had AA in combination with a family history and/or other somatic disease features (constitutional AA), 81 patients with MDS, 123 patients who had features overlapping those of DC, but insufficient for a clinical diagnosis to be made and 111 who were classified as having DC on clinical grounds. The latter were defined as having either (i) the diagnostic triad of mucocutaneous features, or (ii) 1 or more of these features combined with a hypoplastic bone marrow and at least 2 other features known to occur in DC, or (iii) at least 4 of the 6 features commonly associated with the HH syndrome (intrauterine growth retardation, developmental delay, microcephaly, cerebellar hypoplasia, immunodeficiency and bone marrow failure). From this same group of patients we have previously reported on 4 families with MDS/AML and mutations in *TERC* or *TERT* [53], as well as 33 families with mutations in the *TINF2* gene [39], [42] and have also identified 14 families with recurrent *DKC1* mutations and 3 families with recurrent *TERC* mutations.

### Mutation analysis

Genes were scanned for mutation as previously described [43]. Briefly, fragments encompassing all coding exons and flanking intronic sequences of each gene were amplified from genomic DNA by PCR. These fragments were then subjected to denaturing HPLC analysis (in the presence of equal amounts of wild-type DNA fragments in the case of the X-linked *DKC1* gene). Where abnormal patterns of elution were identified, the fragments were re-amplified and sequenced directly using the BigDye™ chain termination method and a 3130xl genetic analyzer (Applied Biosystems).

### Telomere length measurement

Telomere terminal restriction fragment lengths were determined by Southern blot analysis using the sub-telomeric probe pTelBam8 in DNA extracted from total peripheral blood white cells, as previously described [16]. Age adjusted lengths (Δtel) for any one individual were determined by subtracting the expected telomere length, obtained from a regression line drawn from 176 healthy controls, from the observed telomere length as described [54].

We have also used a monochrome multiplex quantitative PCR method to measure telomere length [55] in which the amount of telomeric DNA (T) and the amount of a single copy gene (S) are quantified using standard curves established by titration of a reference genomic DNA sample. All samples were measured in triplicate. T/S ratios, which are proportional to the telomere length [55], were then normalised against a second reference DNA sample that was run on every plate. We have adapted the method for use on a LightCycler 480 real time thermocycler (Roche) as described previously [21].

### Telomerase repeat amplification protocol (TRAP) assay

The TRAP assay was performed as previously described [53]. Briefly, mutations identified in the *TERC* or *TERT* genes were introduced into plasmids harbouring the wild-type gene using a QuikChange Site-Directed Mutagenesis kit (Stratagene). Plasmids were then transfected into WI-38 VA13 cells and after 48 hours the telomerase activity was measured using the TRAPeze RT Telomerase Detection Kit (Millipore/Chemicon).

### Statistical methods

Student's t-tests were performed in ExCel and the Pearson's chi-squred test was performed using VassarStats (http://faculty.vassar.edu/lowry/VassarStats.html).

## Supporting Information

Figure S1 **Sequence traces and location of novel** ***TERC*** **mutations.** Arrows indicate the heterozygous base change named beneath each panel. Their location is shown on a sketch of the TERC molecule.
(PPT)

Figure S2 **Sequence traces and conservation of novel** ***TERT*** **mutations.** Arrows indicate the heterozygous base change named beneath each panel. Alignment of the human, mouse, chicken, frog, yeast and plant TERT protein sequences (generated by MUSCLE at the NCBI) indicates the degree of conservation of the affected amino acid, shown in bold font.
(PPT)

Figure S3 **No correlation between age at report and telomere length.** Age at report versus telomere length of index cases in different genetic subtypes, as indicated on each panel.
(PPT)

Figure S4 **No correlation between number of disease features and telomere length.** Number of disease features versus telomere length in index cases with different genetic subtypes, as indicated on each panel.
(PPT)
