# Supplementary material for: Differences in Disease Severity but Similar Telomere Lengths in Genetic Subgroups of Patients with Telomerase and Shelterin Mutations
Source: PLoS One. 2011 Sep 13;6(9):e24383. doi: 10.1371/journal.pone.0024383 (PMC3172236; doi:10.1371/journal.pone.0024383)
Supplement: Figure S1 — Sequence traces and location of novel TERC mutations. Arrows indicate the heterozygous base change named beneath each panel. Their location is shown on a sketch of the TERC molecule. (PPT) [file pone.0024383.s001.ppt]

## Slide 1
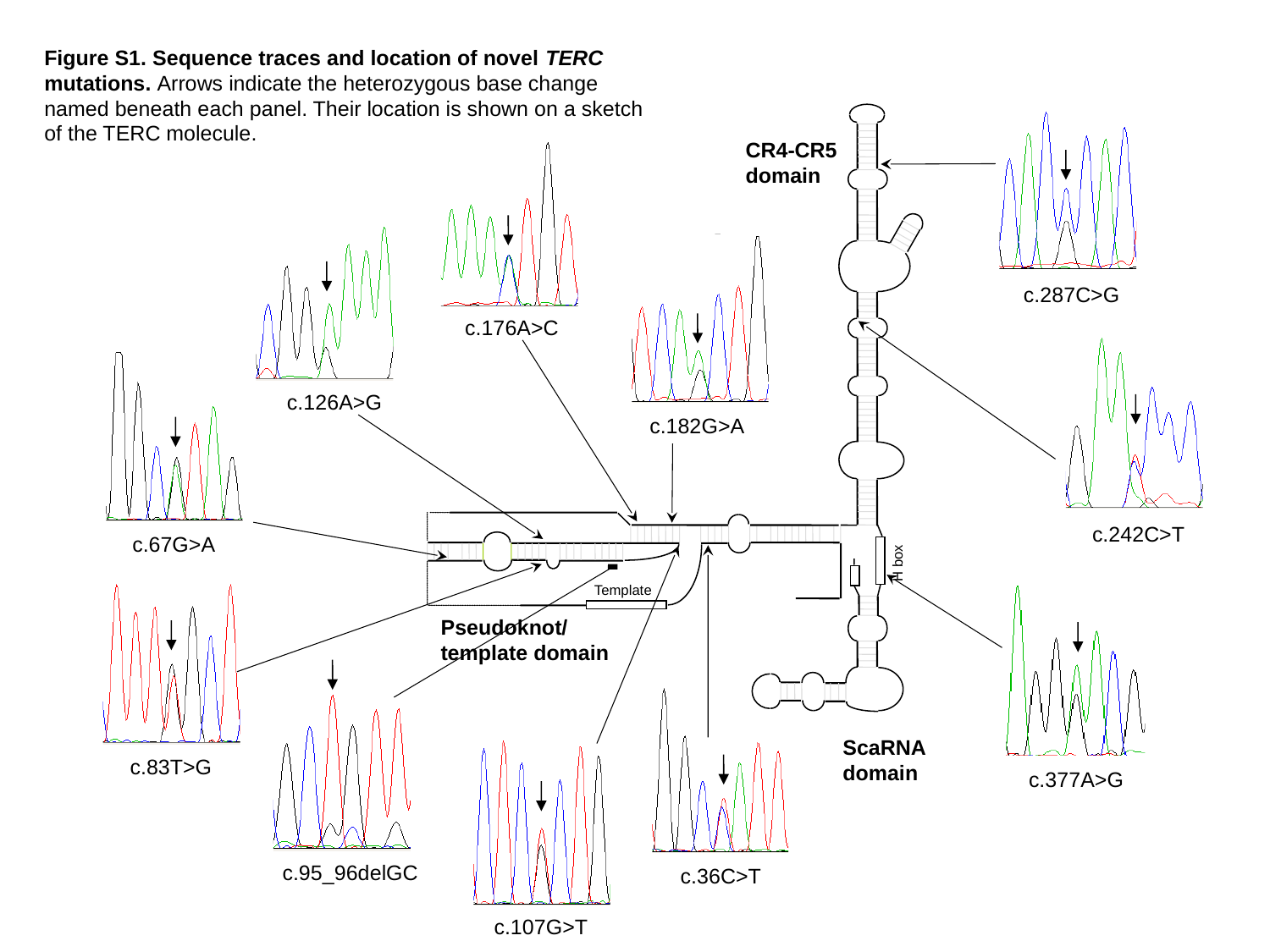

Figure S1. Sequence traces and location of novel TERC mutations. Arrows indicate the heterozygous base change named beneath each panel. Their location is shown on a sketch of the TERC molecule.
CR4-CR5 domain
c.287C>G
c.176A>C
c.126A>G
c.182G>A
c.242C>T
c.67G>A
H box
Template
Pseudoknot/
template domain
ScaRNA
domain
c.83T>G
c.377A>G
c.95_96delGC
c.36C>T
c.107G>T
