# Supplementary material for: Differences in Disease Severity but Similar Telomere Lengths in Genetic Subgroups of Patients with Telomerase and Shelterin Mutations
Source: PLoS One. 2011 Sep 13;6(9):e24383. doi: 10.1371/journal.pone.0024383 (PMC3172236; doi:10.1371/journal.pone.0024383)
Supplement: Figure S2 — Sequence traces and conservation of novel TERT mutations. Arrows indicate the heterozygous base change named beneath each panel. Alignment of the human, mouse, chicken, frog, yeast and plant TERT protein sequences (generated by MUSCLE at the NCBI) indicates the degree of conservation of the affected amino acid, shown in bold font. (PPT) [file pone.0024383.s002.ppt]

## Slide 1
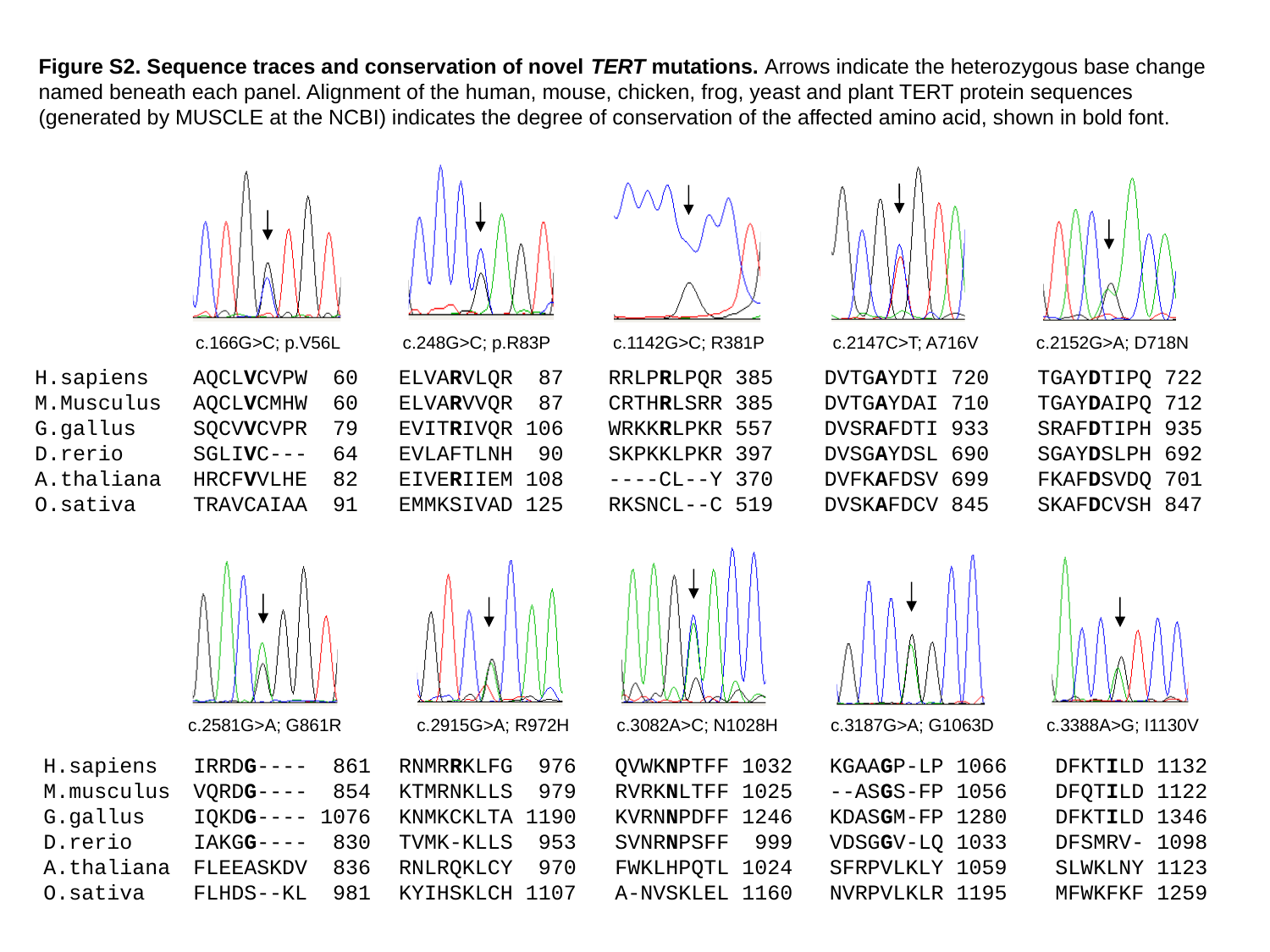

Figure S2. Sequence traces and conservation of novel TERT mutations. Arrows indicate the heterozygous base change named beneath each panel. Alignment of the human, mouse, chicken, frog, yeast and plant TERT protein sequences (generated by MUSCLE at the NCBI) indicates the degree of conservation of the affected amino acid, shown in bold font.
 c.166G>C; p.V56L
 c.248G>C; p.R83P
 c.1142G>C; R381P
 c.2147C>T; A716V
 c.2152G>A; D718N
H.sapiens
M.Musculus
G.gallus
D.rerio
A.thaliana
O.sativa
AQCLVCVPW 60
AQCLVCMHW 60
SQCVVCVPR 79
SGLIVC--- 64
HRCFVVLHE 82
TRAVCAIAA 91
ELVARVLQR 87
ELVARVVQR 87
EVITRIVQR 106
EVLAFTLNH 90
EIVERIIEM 108
EMMKSIVAD 125
RRLPRLPQR 385
CRTHRLSRR 385
WRKKRLPKR 557
SKPKKLPKR 397
----CL--Y 370
RKSNCL--C 519
DVTGAYDTI 720
DVTGAYDAI 710
DVSRAFDTI 933
DVSGAYDSL 690
DVFKAFDSV 699
DVSKAFDCV 845
TGAYDTIPQ 722
TGAYDAIPQ 712
SRAFDTIPH 935
SGAYDSLPH 692
FKAFDSVDQ 701
SKAFDCVSH 847
 c.2581G>A; G861R
 c.2915G>A; R972H
 c.3082A>C; N1028H
 c.3187G>A; G1063D
 c.3388A>G; I1130V
H.sapiens
M.musculus
G.gallus
D.rerio
A.thaliana
O.sativa
IRRDG---- 861
VQRDG---- 854
IQKDG---- 1076
IAKGG---- 830
FLEEASKDV 836
FLHDS--KL 981
RNMRRKLFG 976
KTMRNKLLS 979
KNMKCKLTA 1190
TVMK-KLLS 953
RNLRQKLCY 970
KYIHSKLCH 1107
QVWKNPTFF 1032
RVRKNLTFF 1025
KVRNNPDFF 1246
SVNRNPSFF 999
FWKLHPQTL 1024
A-NVSKLEL 1160
KGAAGP-LP 1066
--ASGS-FP 1056
KDASGM-FP 1280
VDSGGV-LQ 1033
SFRPVLKLY 1059
NVRPVLKLR 1195
DFKTILD 1132
DFQTILD 1122
DFKTILD 1346
DFSMRV- 1098
SLWKLNY 1123
MFWKFKF 1259
