# Supplementary material for: Differences in Disease Severity but Similar Telomere Lengths in Genetic Subgroups of Patients with Telomerase and Shelterin Mutations
Source: PLoS One. 2011 Sep 13;6(9):e24383. doi: 10.1371/journal.pone.0024383 (PMC3172236; doi:10.1371/journal.pone.0024383)
Supplement: Figure S3 — No correlation between age at report and telomere length. Age at report versus telomere length of index cases in different genetic subtypes, as indicated on each panel. (PPT) [file pone.0024383.s003.ppt]

## Slide 1
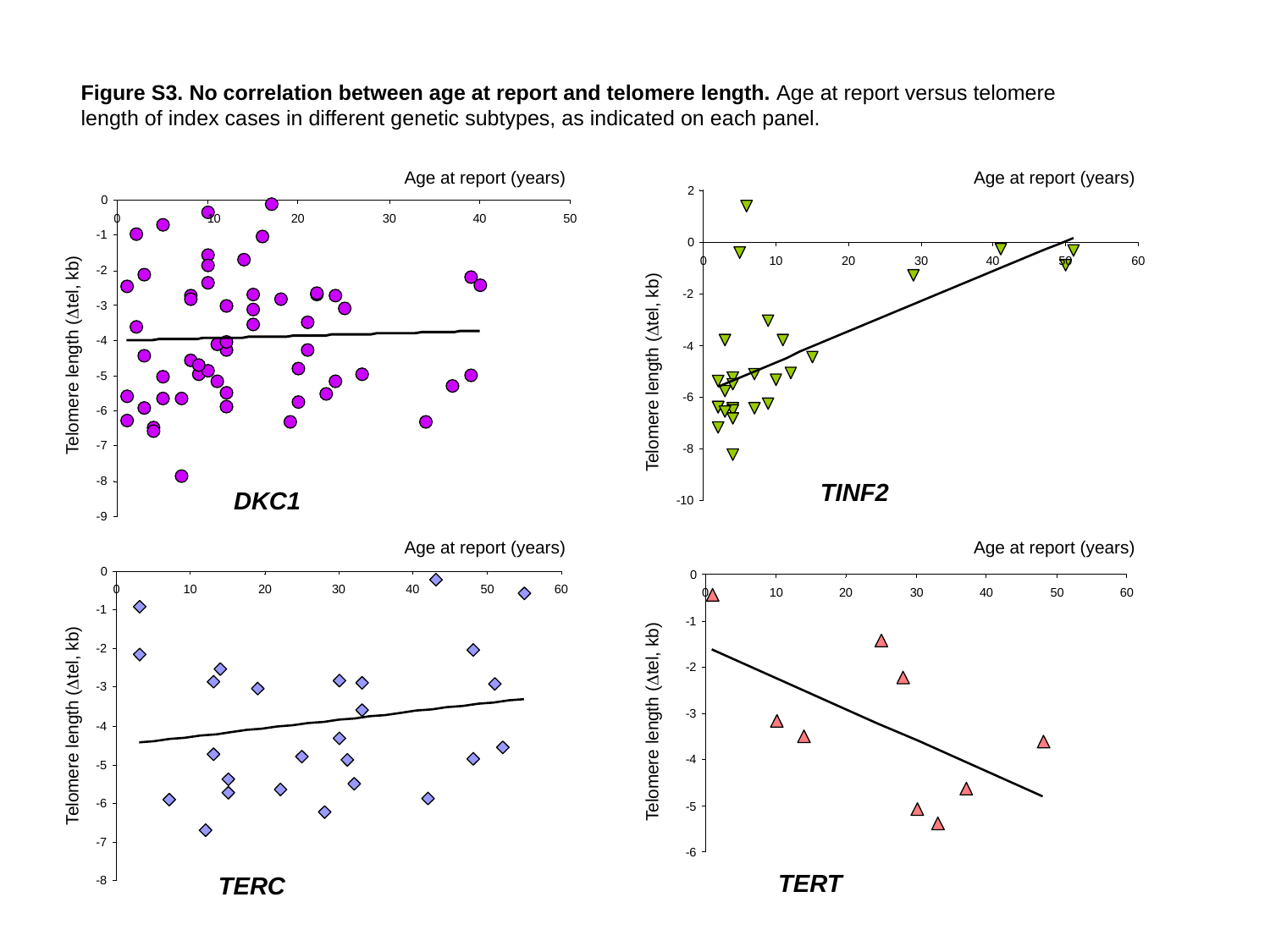

Figure S3. No correlation between age at report and telomere length. Age at report versus telomere length of index cases in different genetic subtypes, as indicated on each panel.
Age at report (years)
Age at report (years)
2
0
0
10
20
30
40
50
-1
0
0
10
20
30
40
50
60
-2
-2
-3
-4
Telomere length (Δtel, kb)
-4
Telomere length (Δtel, kb)
-5
-6
-6
-7
-8
TINF2
-8
DKC1
-10
-9
Age at report (years)
Age at report (years)
0
0
0
10
20
30
40
50
60
0
10
20
30
40
50
60
-1
-1
-2
-2
-3
Telomere length (Δtel, kb)
-3
Telomere length (Δtel, kb)
-4
-4
-5
-6
-5
-7
-6
TERT
TERC
-8
