# Supplementary material for: Differences in Disease Severity but Similar Telomere Lengths in Genetic Subgroups of Patients with Telomerase and Shelterin Mutations
Source: PLoS One. 2011 Sep 13;6(9):e24383. doi: 10.1371/journal.pone.0024383 (PMC3172236; doi:10.1371/journal.pone.0024383)
Supplement: Figure S4 — No correlation between number of disease features and telomere length. Number of disease features versus telomere length in index cases with different genetic subtypes, as indicated on each panel. (PPT) [file pone.0024383.s004.ppt]

## Slide 1
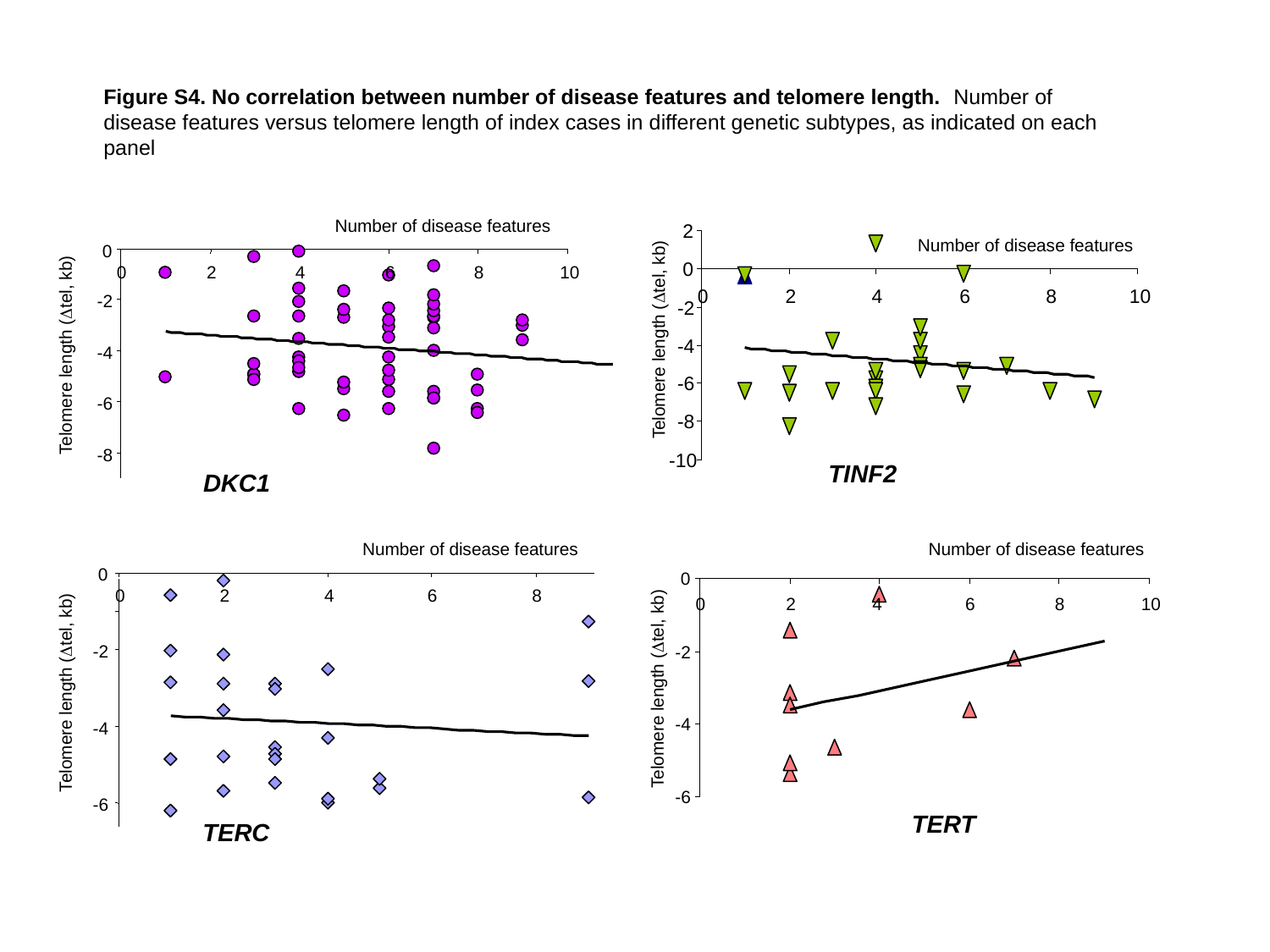

Figure S4. No correlation between number of disease features and telomere length. Number of disease features versus telomere length of index cases in different genetic subtypes, as indicated on each panel
Number of disease features
2
0
0
2
4
6
8
10
-2
-4
-6
-8
-10
TINF2
Number of disease features
0
0
2
4
6
8
10
-2
Telomere length (Δtel, kb)
Telomere length (Δtel, kb)
-4
-6
-8
DKC1
Number of disease features
Number of disease features
0
0
0
2
4
6
8
0
2
4
6
8
10
-2
-2
Telomere length (Δtel, kb)
Telomere length (Δtel, kb)
-4
-4
-6
-6
TERT
TERC
